# Pemphigus vulgaris presenting with epigastric pain

**DOI:** 10.1002/ccr3.7299

**Published:** 2023-05-04

**Authors:** Marawan Elmassry, Jerapas Thongpiya, Pitchaporn Yingchoncharoen, Jali Garza, Matthew Soape, Kanak Das

**Affiliations:** ^1^ Department of Internal Medicine Texas Tech University Health Sciences Center Lubbock Texas USA; ^2^ Department of Gastroenterology Covenant Medical Center Lubbock Texas USA

**Keywords:** autoimmune mucocutaneous disease, epigastric pain, esophageal ulcer, pemphigus vulgaris

## Abstract

Pemphigus vulgaris is an autoimmune mucocutaneous disease with an involvement in gastrointestinal tract especially in oral cavity and esophagus. Ulcers can be the initial presentation even before visible mucosal or cutaneous lesions. The presenting symptoms will be in accordance with the affected organ such as throat pain, hoarseness, dysphagia, odynophagia, or even bleeding. Here, we report a case of undiagnosed pemphigus vulgaris presenting with epigastric pain whose endoscopy showed oropharyngeal involvement and isolate esophageal ulcer, which failed proton pump inhibitor treatment.

## 1 INTRODUCTION

Pemphigus is a group of potentially life‐threatening autoimmune mucocutaneous diseases characterized by epithelial blistering affecting cutaneous and/or mucosal surfaces secondary to IgG antibody synthesis against a desmosome glycoprotein, desmoglein (Dsg), present in keratinocytes. Its estimated incidence is at two new cases/millions/year. Most are seen in the fifth and sixth decades of life with rare cases seen in children and the elderly.^1^ Although there are variants within pemphigus, pemphigus vulgaris accounts for 80% of cases. There are two clinical forms of pemphigus vulgaris: mucosal (MPV) and mucocutaneous (MCPV) which patients' mucosa is affected and over five skin lesions are present.^2^ The recent study showed that patients with pemphigus vulgaris have oral lesion about 90% with solely oral mucosal lesion around 50%.^3^ The presentation symptoms include tenderness in the throat, pain on swallowing, hoarseness, or dysphonia.^2^ The authors present a case of pemphigus vulgaris with oropharyngeal and esophageal involvement presenting uniquely with an epigastric pain.

## 2 CASE REPORT

**FIGURE 1:**
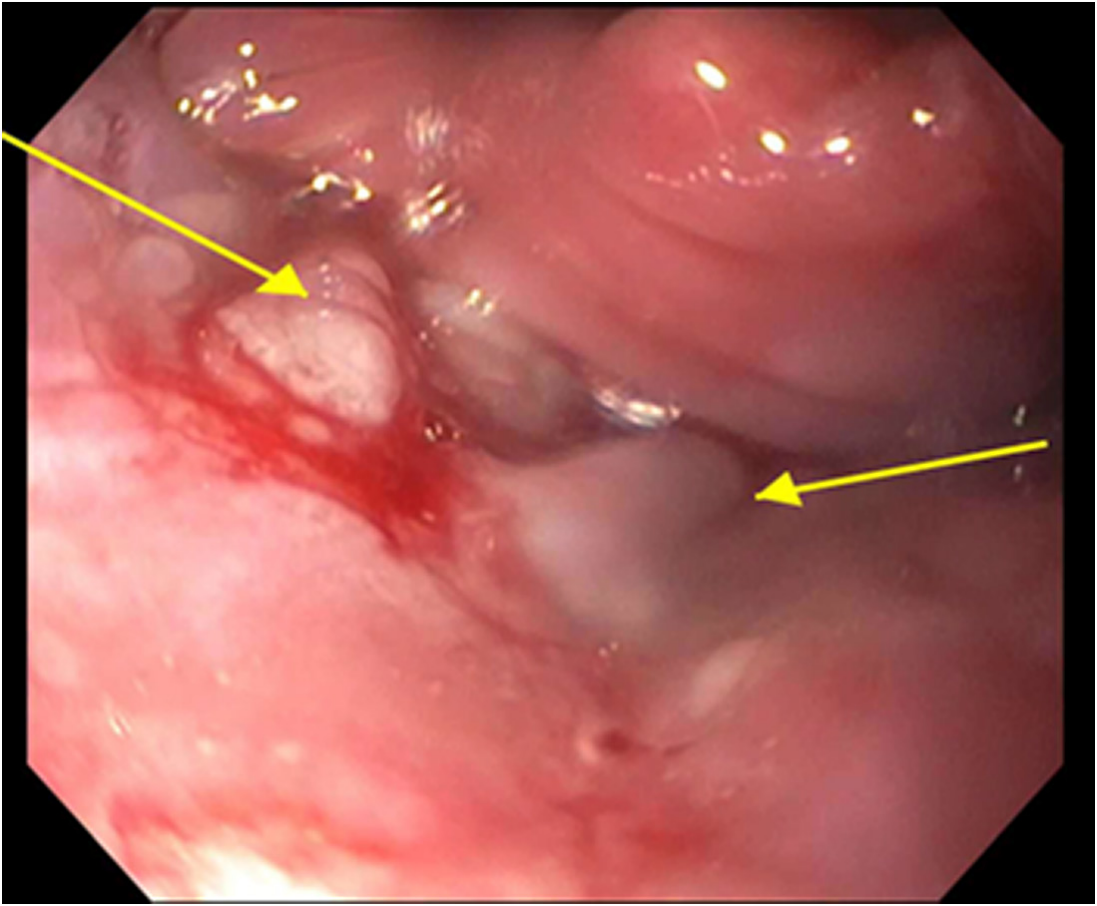
Bleeding esophageal ulcer just below the upper esophageal sphincter.

**FIGURE 2:**
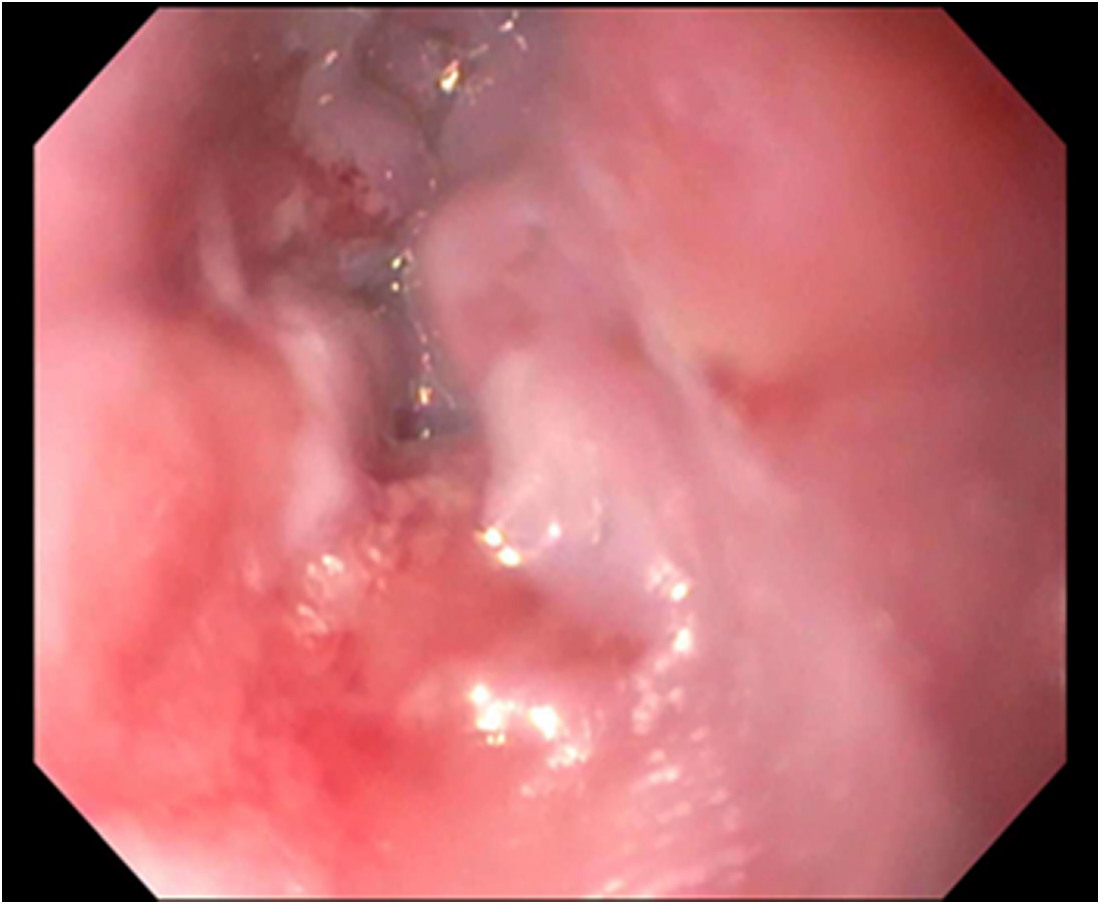
Nodularity and inflammation above the upper esophageal sphincter.

A 59‐year‐old female with past medical history of hypertension presented to the hospital with severe epigastric pain radiating to the neck for 4 days. Recently the patient had been prescribed with pantoprazole daily with titration to twice daily for 2 months for her epigastric pain with no relief. Initial labs including CBC, CMP, throat swab, and culture were unremarkable. Computed tomography (CT) soft tissue neck showed possible tonsilitis. Gastroenterology was consulted and patient underwent esophagogastroduodenoscopy (EGD) that revealed a single esophageal ulcer in the upper esophagus with subsequent benign biopsies. Patient was managed with pain control and continued pantoprazole once per day. At her outpatient follow‐up visit, she complained of continued pain and weight loss due to inability to eat. 1 month after the initial hospital stay, the patient returned to the ED with worsening symptoms. EGD was repeated at this point with similar finding of one superficial esophageal ulcer with oozing blood in the proximal esophagus just below the upper esophageal sphincter (Figure 1). Biopsies once again revealed benign esophageal ulcer. There was also noted to be inflammation and nodularity in the posterior pharynx below the vocal cords (Figure 2). At this point ENT was consulted, and it was decided to perform biopsies of the tongue and soft palate. Biopsy results were consistent with a diagnosis of oral pemphigus vulgaris. The patient was started on steroid therapy and saw resolution of her epigastric pain. She continues low dose prednisone every day for maintenance therapy 6 months after diagnosis.

## 3 DISCUSSION

The evolution of PV typically begins with painful mucosal ulceration, especially in the mouth. These ulcers may be persistent or intermittent, but the new lesion will continue to appear. Many patients will develop skin lesions over the following weeks or months with an average of 5 months. Pemphigus vulgaris can affect any site of oral mucosa which gingival papillary tips are common. White patches of hyperkeratosis can be seen after PV heals.^4^ In fact, esophageal involvement has a relatively high incidence ranging from 46 to 87%, but EGD was done infrequently enough to detect.^5^ Esophageal involvement in pemphigus vulgaris usually presents with throat pain, dysphagia, odynophagia or even bleeding. However, patient with oropharyngeal and upper esophageal involvement presented with an epigastric pain is very uncommon.

Espana et al.^2^ found that PV patients usually have active lesions in both pharyngeal and laryngeal mucosa leading to throat, pharyngeal, or laryngeal symptoms.

Nakamura et al.^6^ found that esophageal involvements of PV include blisters, erosions, erythema, scattered red spots, and longitudinal erythematous lines with positive Nikolsky sign, mostly in the upper esophagus. The distribution of lesions can be whole esophagus with more severity in proximal than distal or more rarely isolated lesion.

In our patient, she presented with epigastric and neck pain, which might not be a straightforward manifestation of PV, but can be explained by her oropharyngeal involvement. Her isolate esophageal ulcer found on EGD with benign biopsy initially did not lead us to the diagnosis but with retroflexion of EGD, the inflammation of pharynx helped further identify the clues to diagnosis. In addition, the friable ulcer with bleeding as seen here could also be considered as a positive Nikolsky sign. Early diagnosis of the disease becomes very important as the disorder can be life‐threatening. Once a diagnosis is made, treatment should be initiated.

Management of PV is considered in two main phases: remission induction (to achieve disease control with no new lesion formation and existing ones to be healed) and remission maintenance (disease‐free for at least 2 weeks). First‐line treatment includes corticosteroid, usually in form of oral prednisolone (0.5–1 mg/kg/day) and start tapering at the end of remission maintenance. It is also thought to lower dosing and duration of steroid therapy in patients which allows for lesser risk of adverse side effects of therapy. Second‐line treatment indicated if treatment failure occurs and it includes azathioprine, mycophenolate mofetil, cyclophosphamide, or methotrexate. Rituximab is recently approved as a third‐line treatment.^4^

## 4 CONCLUSION

It is important to keep in mind that a patient with pemphigus vulgaris and oral involvement can present with an unusual presentation such as an epigastric pain like this patient.

## CONSENT

Written informed consent was obtained from the patient to publish this report in accordance with the journal's patient consent policy.

## AUTHOR CONTRIBUTIONS

**Marawan Elmassry:** Writing – original draft. **Jerapas Thongpiya:** Writing – original draft. **Pitchaporn Yingchoncharoen:** Writing – original draft. **Jali Garza:** Data curation. **Matthew Soape:** Investigation. **Kanak Das:** Writing – review and editing.

## CONFLICT OF INTEREST STATEMENT

The authors have no financial conflicts to disclose.

## Data Availability

Data openly available in a public repository that issues datasets with DOIs.

## References

[1] Porro AM , Seque CA , Ferreira MCC , Enokihara MMS . Pemphigus vulgaris. An Bras Dermatol. 2019;94:264‐278. 31365654 10.1590/abd1806-4841.20199011 PMC6668932

[2] España A , Fernández S , Del Olmo J , et al. Ear, nose and throat manifestations in pemphigus vulgaris. Br J Dermatol. 2007;156(4):733‐737. doi:10.1111/j.1365-2133.2007.07783.x 17493073

[3] Batistella E , Sabino da Silva R , Rivero ERC , Silva CAB . Prevalence of oral mucosal lesions in patients with pemphigus vulgaris: a systematic review and meta‐analysis. J Oral Pathol Med. 2021;50(8):750‐757. doi:10.1111/jop.13167 33713362

[4] Melchionda V , Harman KE . Pemphigus vulgaris and pemphigus foliaceus: an overview of the clinical presentation, investigations and management. Clin Exp Dermatol. 2019;44(7):740‐746. doi:10.1111/ced.14041 31378971

[5] Okamura A , Nakamura R , Yamagami J , et al. Evaluation of pharyngo‐oesophageal involvement in pemphigus vulgaris and its correlation with disease activity. Br J Dermatol. 2017;176(1):224‐226. doi:10.1111/bjd.14725 27167757

[6] Nakamura R , Omori T , Suda K , et al. Endoscopic findings of laryngopharyngeal and esophageal involvement in autoimmune bullous disease. Dig Endosc. 2017;29(7):765‐772. doi:10.1111/den.12893 28475223

